# Chemists Debate the Value of Elemental Analysis

**DOI:** 10.1021/acscentsci.2c01438

**Published:** 2022-12-14

**Authors:** Alla Katsnelson

A few years ago, Saurabh Chitnis, a synthetic chemist at Dalhousie University, did some math that spurred a radical decision. He calculated what it was costing him to send lab-made compounds away for elemental analysis, a classic technique in which a compound is burned to determine its molecular composition. Then, fueled by that enormous sum, he sat down and wrote a grant to buy a $75,000 machine. Installed last September, it allows him and six other chemists in his department to do the analysis on-site.

“I’m not an analytical chemist who would typically have this instrument,” Chitnis says. “It’s very specialized and expensive, not just to purchase but also to maintain.” But his calculations showed it was worth it. “At one point, 12% of my research funding was going towards burning my chemistry.”

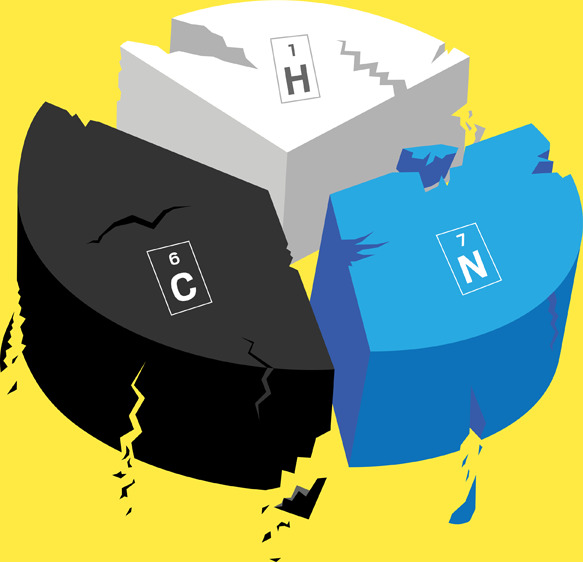



These days, chemists who need to conduct elemental analysis often have to send their compounds to companies or to other universities that do elemental analysis for a fee. For each sample, these testing companies typically send back three numbers, without the raw data to back them, indicating what percentage of the burned sample consists of carbon, hydrogen, and nitrogen.

Despite the cost of elemental analysis, Chitnis needs these data if he wants to publish his work. Many referees demand elemental analysis data before recommending a paper for publication, stipulating that values can deviate from the compound’s formula by 0.4% to be deemed sufficiently pure for publication.

The problem—and one of the reasons behind Chitnis’s purchase—is that results from commercial providers can be wildly inconsistent, likely because of differences in calibration across instruments and possibly a lack of expertise.

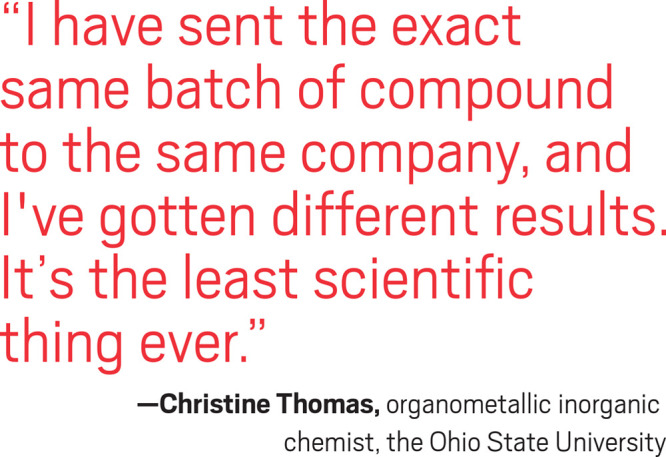



“I have sent the exact same batch of compound to the same company, and I’ve gotten different results,” says Christine Thomas, an organometallic and inorganic chemist at the Ohio State University. “It’s the least scientific thing ever.”

Similar tales abound of chemists repeatedly sending samples until they get the numbers they need for their publications. There are other issues too. Having an arbitrary blanket requirement of 0.4% for all compounds does not make sense, say some chemists who spoke with *C&EN*, and for some studies, the compound’s purity is not as relevant as it is for others. Yet journals or reviewers sometimes see the requirement as absolute, with no room for leeway.

Some chemists argue that the time has come for the chemistry community to reassess exactly when elemental analysis is relevant and how purity should be proven. Two studies in the past 2 years—one of them by Chitnis and colleagues—have detailed foibles in the use of elemental analysis and point to ways that journals’ approaches to purity standards could change. “It is a long-standing issue in the community, a pain point for generations of synthetic chemists, for sure,” Chitnis says.

## The fundamentals of elemental analysis

Elemental analysis is a straightforward technique: it involves heating a compound until it combusts and then burning it fully until the carbon, hydrogen, and nitrogen oxidize to carbon dioxide, nitrogen oxides, and water. Chemists can backtrack from the quantities of those combustion products and calculate the percentages of carbon, hydrogen, and nitrogen in the sample. Sample purity is gauged by how well those percentages align with what’s expected for a compound.

French chemist Antoine Lavoisier invented elemental analysis in the late 1700s, and it quickly became a key method. For example, Danish chemist William Christopher Zeise used it to determine the composition of the organoplatinum compound that is now called Zeise’s salt, says Ulrich Schatzschneider, a bioinorganic chemist at Julius Maximilian University of Würzburg.

Until about the 1960s, elemental analysis was the only way to determine a synthesized compound’s composition. Back then, elemental analyzers—and staff with the expertise to run them—were common in university facilities. With the rise of newer analytical techniques such as nuclear magnetic resonance and mass spectrometry, which in many cases provide more sophisticated information, many institutions have eliminated on-site elemental analysis.

But NMR and mass spec have limitations. NMR, for example, can detect only certain elements and struggles with impurities like sodium chloride. And mass spectrometry’s detection abilities depend on how a compound ionizes. “If you have a molecule that is already charged, you’ll probably be able to see the parent ion, but if your molecule is neutral, who knows what fragments you’re going to see,” Thomas says. Plus, mass spec will not detect impurities with low mass, like NaCl and most organic solvents, says Christian Kowol, an inorganic chemist at the University of Vienna. Meanwhile, elemental analysis is frequently seen as a reliable tool for sussing out these stray molecules.

But when faced with inconsistent results from commercial analysis—especially when evidence from other techniques is clear or when a compound’s purity is not the paper’s point—chemists wonder if elemental analysis is worth the time and money. And when the results are off, “we weren’t sure what problem we were supposed to fix,” Chitnis says. “Should we package the compounds better, purify them, or is the calibration of the instrument on the other end wrong?” *C&EN* contacted several elemental analysis providers for this story but did not hear back by its deadline.

## Putting elemental analysis to the test

In the face of this uncertainty, chemists have taken it upon themselves to validate the precision of third-party providers of elemental analysis. In June, Rebecca Melen of Cardiff University published a study co-led by Chitnis, Caleb D. Martin of Baylor University, and Jason L. Dutton and Rupert E. H. Kuveke of La Trobe University to examine whether the 0.4% standard was realistic.

For the study, they bought five organic compounds and determined that they fell within the 0.4% benchmark on Chitnis’s instrument, which had been calibrated last summer. Then the researchers sent the compounds to 17 facilities across three continents. A significant portion of the results—16.44% of the tests for carbon and 13.89% of the tests for nitrogen—failed to hit 0.4%.

These data, along with concerns of variability in how providers handle samples, mean meeting the benchmark for purity is not in a scientist’s control. That makes the 0.4% metric “not a scientifically justifiable standard,” the researchers write in a recent commentary in *Chemistry World*. What’s more, the rationale for that value is nowhere to be found, and no one seems to know who established it and when. “It’s just given to us like the tablets of Moses,” Chitnis says.

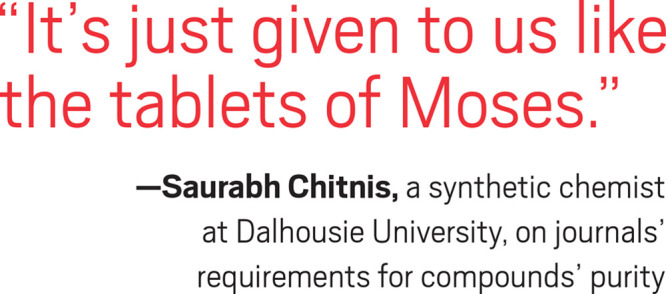



A lack of confidence in third-party providers may be leading people to take shortcuts. Because journals do not require any supporting documentation for elemental analysis results, researchers might be tempted to fudge their numbers, Schatzschneider says.

“To be frank, I know that some people commit fraud,” says Vincent LaVallo, a synthetic chemist at the University of California, Riverside. Ideally, he says, researchers would do it themselves, at their own institutions, same as with other data they gather, rather than pay an outside facility to do it until they get the right numbers back. “Really, it comes down to, ‘Why do we have so much faith in these third parties, who have a financial stake in the outcome, to perform this?’ ”

Funding agencies are increasingly requiring that raw data be made publicly available, says Melen. “So I find it surprising that with elemental analysis you just have to trust the numbers you’re given by wherever you send the sample off to.”

Last year, Kowol, Wolfgang Kandioller, and their colleagues at the University of Vienna published a study showing that sometimes impurity values reported for elemental analysis are so low that they are unlikely to be real.

First the researchers scanned the literature, and they estimated that in about 5–10% of papers, most or all of the values were below 0.1% impurity. Then as an experiment to see how these values stacked up against real-world results, the team had six instruments analyze three organic compounds and three metal complexes, all freshly opened. Some of the instruments were at the researchers’ universities, and some were at three companies. Most of these measurements deviated from the theoretical values by 0.05–0.20%.

In light of these results, Kowol says, consistently getting sub-0.1% deviations from theoretical values—particularly when a paper reports such values for five or more compounds—is extremely unlikely. “When you have some experience with chemistry and elemental analysis over the years, you look at them and you say, ‘OK, that’s simply impossible.’ ”

Still, despite the benchmark’s unknown provenance, Kandioller says, the number makes sense. Given that most bioinorganic and many organic compounds contain about 50% carbon plus or minus 10 percentage points, he explains, a result within 0.4% would amount to at least 98% purity. He and Kowol note that in their study, the benchmark was not difficult to obtain, even with commercial testers.

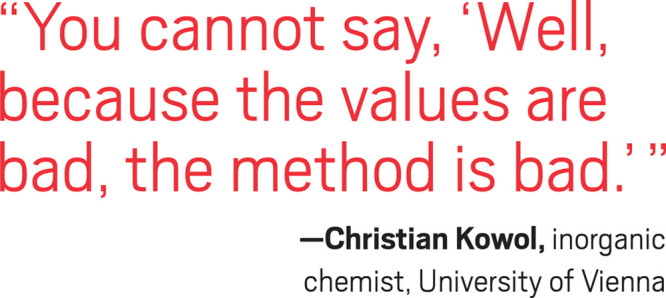



They say that while many companies may make errors, that’s not a scientific rationale for changing the way the technique is used. “When you have inexperienced technicians, you cannot say, ‘Well, because the values are bad, the method is bad,’ ” Kowol says. Particularly concerning, Kandioller adds, is the fact that authors are now referencing Melen’s study when the elemental analysis on their compounds does not hit the target. “The paper is now an excuse not to care about the values,” Kandioller says.

For their part, Melen and Chitnis are glad that people can use their paper to get reviewers to be more flexible.

## Change wanted

Despite their differences, chemists, including the authors of the Kowol- and Melen-led papers, widely agree that resending compounds multiple times until researchers get the “correct” value—the equivalent of throwing noodles against the wall until they stick—is bad for the field. Proponents of loosening the 0.4% standard argue that keeping it wrongly implies it is always achievable. Meanwhile, those who support maintaining the standard say that deviations bigger than 0.4% likely reflect actual impurities.

Either way, Kowol notes that a requirement by journals to include the raw data in a paper’s supporting information would make researchers less tempted to falsify numbers. In their study, he and his colleagues recommend that authors include details of instruments used and their calibration, as well as chromatograms for each sample in papers’ methods sections. That would require obtaining these data from companies, but if journals demanded these materials, companies would adapt, Kowol says.

Melen and her colleagues agree that such information should be included. In their paper, though, they argue that changing the impurity cutoff to 0.7% would mean 95% of the compounds they analyzed would fall into the acceptable range.

Slowly, for better or for worse, the cachet of elemental analysis within the chemistry community is changing. Both Kowol and Melen say that editors at multiple journals have contacted them to discuss possible changes suggested by their studies. Some journals are beginning to soften their position, reframing the strict requirement as more of a guideline. And recently, for example, *Angewandte Chemie* and the *European Journal of Inorganic Chemistry* removed the 0.4% requirement from their guidelines.

When Paul Chirik, Editor in Chief of the American Chemical Society journal *Organometallics*, was asked about the journal’s requirements, he pointed to a 2016 editorial explaining a shift toward a more lenient policy. In that editorial, he and six other authors wrote, “We envisage a system where elemental analysis is no longer an inflexible requirement, but rather very strongly encouraged.” (ACS publishes *C&EN* and *ACS Central Science*.)

Other journals, such as *Chem*, also stipulate some flexibility, noting that authors should “ideally” include elemental analysis results that fall within the 0.4% of theoretical numbers for small molecules. Elemental analysis is an important method, says Vjekoslav Dekaris, senior scientific editor at *Chem*, but “analytical chemistry has evolved to a degree that elemental analysis is not the sole key tool to decipher sample identity and ensure sample purity anymore.”

It makes sense that changes are moving slowly, Melen says. She and Chitnis hope that other researchers might explore elemental analysis’s role by looking at larger sample sets and different classes of compounds that are air sensitive or that contain a large diversity of elements. If the 0.4% standard should be changed, for example, what should the new standard be, and should the value differ for the three? Such questions should be answered through further studies, she says. “There’s so much more that could be done.”

*Alla Katsnelson is a freelance contributor to**Chemical & Engineering News*, *the independent news outlet of the American Chemical Society.*

